# Challenges of Managing Lower Urinary Tract Symptoms in Women with Tamoxifen Use

**DOI:** 10.1089/whr.2021.0147

**Published:** 2022-04-11

**Authors:** Ethan M. Fan, Philippe E. Zimmern

**Affiliations:** Department of Urology, University of Texas Southwestern Medical Center, Dallas, Texas, USA.

**Keywords:** tamoxifen, urinary tract infection, urinary incontinence, pelvic organ prolapse, women

## Abstract

**Objective:**

Tamoxifen complicates management of conditions such as urinary tract infections (UTIs), urinary incontinence (UI), and/or pelvic organ prolapse (POP) that traditionally benefit from hormonal intake; thus, we reviewed our experience in managing these hormonally deprived women.

**Materials and Methods:**

After IRB approval, electronic medical records from women with current use or history of tamoxifen use and referred to a tertiary care center with female pelvic medicine and reconstructive surgery expertise for UTI, UI, and/or POP were reviewed.

**Results:**

From 2015 to 2020, 32 women treated with tamoxifen 10–40 mg for a median of 4 years were referred for UTIs (9), UI (10), symptomatic POP (8), or for a combination of these (5). Participants with UTI treated with antibiotics, prophylactic supplements, and/or electrofulguration had satisfactory response at median follow-up of 1 year (interquartile range [IQR]: 0.5–1). Ten of 15 women with UI chose intervention, with no self-reported UI recurrence at median follow-up of 2.5 years (IQR: 1–3). All but one participant with POP underwent vaginal or open/robotic mesh repairs, with satisfactory outcomes at median follow-up of 3 years (IQR: 2–7).

**Conclusions:**

The management of UTIs, UI, and POP in women on tamoxifen or unable to benefit from hormonal intake is challenging, but traditional interventions can be considered with satisfactory results.

## Introduction

Tamoxifen is commonly used for treatment and adjuvant therapy of estrogen receptor-positive breast cancers but is also indicated for risk reduction in high-risk women or those with ductal carcinoma *in situ*.^1–5^ Categorized as a selective estrogen receptor modulator, tamoxifen binds to estrogen receptors and acts as an agonist in some tissues and antagonist in others.^1,4^ The mechanism of tamoxifen's action on the vaginal wall is not fully known. Clinical findings of dyspareunia and vaginal dryness after tamoxifen exposure indicate a primarily antagonistic effect on the vagina^6–8^; however, studies, as summarized in Table 1, on the histological effects of tamoxifen on the vagina in postmenopausal women show a partial estrogen agonist role, possibly suggesting a shift in receptors with age.

**Table 1.** Literature Review on the Effect of Tamoxifen on the Vagina

Author and year	No. of women	Population	Tamoxifen dosage and duration	Findings
Ferrazzi et al. 1977^38^	35	PM with late breast cancer resistant to previous endocrine treatment and chemotherapy	30–40 mg daily for 30–45 days	□ KPI in vaginal smears (measure of estrogen activity) increased after starting tamoxifen □ Smears atrophied within 2 months of tamoxifen withdrawal
Boccardo et al. 1981^39^	32	PM after radical mastectomy for breast cancer	10 mg twice a day	□ KPI in vaginal smears while on tamoxifen increased
Eells et al. 1990^40^	10	PM with breast cancer	10 mg twice a day	□ Maturation value (measure of estrogen effect) increased in 70% of Pap smears after starting tamoxifen □ No correlation between time on tamoxifen and estrogen effect
Lahti et al. 1994^41^	46 in tamoxifen group, 45 in control group	PM with breast cancer	20–40 mg daily for median 32 months	□ Estrogenic effects seen in Pap smears in 89% of tamoxifen participants versus 49% of control
Sobel et al. 1996^46^	3	PM with breast cancer	1–7 years	□ Development of vulvovaginal candidiasis while on tamoxifen (rare in this population otherwise)
Mortimer et al. 1999^42^	57	With breast cancer	2 months to 2 years	□ High maturation index (measure of estrogen effect) seen on over two-third of vaginal smears while on tamoxifen □ Estrogen effect associated with vaginal dryness and dyspareunia (measured by questionnaire)

KPI, Karyopyknotic index; PM, postmenopausal.

A current use or history of tamoxifen use complicates the management of urological conditions, particularly urinary tract infections (UTIs), urinary incontinence (UI), and pelvic organ prolapse (POP) as it relates to vaginal estrogen.^9–11^ Vaginal estrogen is avoided due to fear of systemic absorption, which could increase the risk of recurrence of estrogen receptor-positive breast cancer. Guidelines by ACOG (American College of Obstetricians and Gynecologists) recommend use of nonhormone treatments as first line for treatment of urogenital symptoms in women with a history of breast cancer.^12^

There is a gap of knowledge on long-term management of UTIs, UI, and POP in women with a history of tamoxifen use. It is unknown whether tamoxifen affects the vagina such that it impacts the success rate of nonhormonal medical and surgical interventions. There is also a lack of knowledge regarding development of side effects affecting the lower urinary tract from tamoxifen use.

Women on tamoxifen are often told that their inability to receive hormone therapy will compromise their pelvic care; therefore, since few are referred for management, there is a dearth of data on how these women fare long term when treated conventionally. Thus, this study reviewed the efficacy of nonestrogen management for UTIs, UI, and POP alone or in combination, in women with current or previous tamoxifen exposure, to determine whether such exposure influenced their management outcomes.

## Materials and Methods

After receiving IRB approval, a retrospective chart review was performed on women who had current exposure or history of tamoxifen exposure and were referred to a single female pelvic medicine and reconstructive surgery (FPMRS) tertiary care urology clinic over the past 5 years. Participants were excluded from analysis if their reason for referral did not include UTI, UI, or POP and if follow-up after management was <6 months.

Demographics, reason for referral, urological history and management, and tamoxifen history were collected from electronic medical records (EPIC). Tamoxifen history collected included dosage, duration, side effects, reason for stopping (if not currently taking), and current breast cancer status. All women referred for UTIs were evaluated as per current guidelines with upper tract studies, office flexible cystoscopy, and standing voiding cystourethrogram (VCUG)^13^ to determine whether there were any underlying conditions causing their UTIs, such as urinary reflux, urinary retention, bladder prolapse, or kidney stones.

Data on UTI frequency, urine cultures, UTI complications (such as pyelonephritis or recurrence), and treatment (particularly supplements, antibiotics, and electrofulguration^14^) were collected. UTI episodes were counted if there was documented treatment for a positive urine culture. History of UTIs before referral also included episodes reported by the participant or notes from an outside facility. For women with UI and/or POP, data on urodynamic studies (UDSs), imaging, and treatment (particularly surgical procedures) were collected. Follow-up data extended to the last interaction with the participant by office visit, MyChart message, or phone call.

Response to UTI treatment was successful if the rate of UTIs after treatment was less than two UTIs in 6 months or three UTIs in a year, the cutoff for recurrent UTIs.^15^ For those undergoing electrofulguration, success was also predicated on a 6-month postfulguration office cystoscopy showing the complete resolution of superficially cauterized lesions and no appearance of new inflammatory lesions in the bladder. UI treatment outcomes were based on the short forms of validated symptom questionnaires Urinary Distress Inventory (UDI-6) and Incontinence Impact Questionnaire (IIQ-7).^16^

Based on a study by Skorupska et al.,^17^ response was marked as successful if the questionnaire total scores during follow-up visits were less than those before treatment, the UDI-6 total score was <33.33 post-treatment, the IIQ-7 total score was <9.52 post-treatment, and the follow-up notes did not record any abnormal physical examination findings or surgical complications. Response to treatment for POP was noted on physical examination and POP quantification (POP-Q) staging,^18^ with success marked if there was improvement to prolapse stage 0 or 1 after treatment, and there were no complaints of symptoms including vaginal bulge from the participant. Any recurrence of the original condition or complications during the follow-up period were noted.

Descriptive statistics were reported with median and ranges for continuous variables and frequencies for categorical variables.

## Results

Thirty-two of 41 women met study criteria, of which 10 had previous and 22 had current exposure to tamoxifen at time of follow-up. Six of the nine excluded women were referred for urinary frequency, urethral pain, incomplete emptying, removal of vaginal foreign body, and vaginal discharge unrelated to UTI, UI, and POP. The other three were excluded due to insufficient follow-up. Out of 27 who presented with a single condition, 9 were for UTIs, 10 for UI, and 8 for symptomatic prolapse. Five had more than one condition: UTI with UI (one) and UI with POP (five). Demographic data are presented in Table 2. Median age was 73 years (interquartile range [IQR]: 66.75–80.5), and majority were White (76%).

**Table 2.** Participant Characteristics

	*n* = 32
Median age, years, *n* (IQR)	73 (66.75–80.5)
Race, *n* (%)	
White	13 (76)
Black/African American	2 (12)
Hispanic	1 (6)
Asian	1 (6)
Median BMI, *n* (IQR)	25.2 (22.9–29.4)
Diabetes, *n* (%)	4 (13)
Median gravidity, *n* (IQR)	2 (1–3)
Median parity, *n* (IQR)	2 (1–3)
Hysterectomy, *n* (%)	25 (78)
Menopause, *n* (%)	
Bilateral salpingo-oophorectomy	19 (59)
Natural menopause	13 (41)
Sexually active, *n* (%)	10 (31)
Smoker, *n* (%)	
Never	19 (61)
Former	10 (32)
Current	2 (6)
Tamoxifen history, *n* (%)	
Current	22 (69)
Previous	10 (31)

BMI, body mass index; IQR, interquartile range.

Only 10 were sexually active, and among those, there was no reported use of vaginal moisturizers or lubricants. All women were treated with a dose of 10–40 mg of tamoxifen. Although typical treatment with tamoxifen lasts at least 5 years,^19^ most of the women who had a history of tamoxifen use were discontinued from their tamoxifen course earlier than expected by gynecological oncology, resulting in an overall median treatment duration of 4 years (IQR: 1–5).

The reason for discontinuing tamoxifen was not recorded in many of participants' charts, but the most common reason given was side effects, which included menopausal symptoms such as hot flashes (six), postmenopausal bleeding (one), and transaminitis (one). For women with previous tamoxifen use, median time from last tamoxifen use and follow-up was 3 years (IQR: 1–4). Only four participants were still on tamoxifen at the time of data extraction. All women not currently on tamoxifen were in remission from breast cancer, aside from two who relapsed.

Of the 10 women referred for UTIs, 5 were started on daily prophylactic medications (low-dose antibiotics, d-mannose, and/or methenamine), 2 were given self-start antibiotics, and 3 were prescribed antibiotics. Three participants with extensive lesions of chronic trigonitis, RUTI episodes, and no response to multiple antibiotic courses elected to undergo superficial electrofulguration with adequate subsequent healing and no recurrence of cystitis on cystoscopy at 6 months. On work-up, no participants had any underlying urological conditions that increase the risk of UTIs (reflux, retention, *etc*.). All showed improvement of symptoms as well as no incidence of pyelonephritis, sepsis, and hospitalizations for UTIs at a median follow-up of 1 year (IQR: 0.5–1).

Of 15 women referred for UI, 10 chose intervention, all of whom had no UI recurrence at a median follow-up of 2.5 years (IQR: 1–3). Four opted for surgical interventions (one underwent Macroplastique^®^ injections^20^ (Minnetonka, MN, USA), two had fascial slings,^21,22^ and one had a Burch procedure^21,22^) based on stress UI severity by UDS findings^23,24^ and degree of prolapse by examination and confirmed by standing VCUG.^25^ Two participants with primarily urge UI were prescribed medications (oxybutynin and/or mirabegron). Four underwent pelvic floor physical therapy, along with behavioral bladder modifications. The remaining five participants with UI decided to pursue only behavioral modifications: three reporting a decrease in symptoms and two reporting no change at 6 months or more.

All 12 women with POP initially had a stage of 2 or 3 and underwent surgical procedures except for one who was treated with pessary. Surgical treatment was successful on physical examination, including POP-Q points, and no reported symptoms of recurrent vaginal bulge at a median follow-up of 3 years (IQR: 2–7). Seven procedures were performed vaginally (anterior vaginal wall suspension,^26^ rectocele repair, and/or complete prolapse repair).^27^ Four were open/robotic mesh repairs (sacrocolpopexy^28,29^), two of which were posthysterectomy.

## Discussion

This limited observational study aimed to examine the nonhormonal management of three frequent conditions in postmenopausal women currently or previously exposed to tamoxifen. Despite lack of estrogen use, their respective treatment for UTIs, UI, and/or POP produced satisfactory outcomes consistent with existing data in women not exposed to tamoxifen and benefitting from estrogen supplementation. This is a very encouraging finding for those worried about tamoxifen negatively affecting the management of their pelvic floor symptoms related to UI, POP, and/or recurrent UTIs.

Vulvovaginal atrophy, a condition that is prevalent in ∼50% of postmenopausal women, is caused by a lack of estrogen in vaginal tissue leading to thinning and dryness of the vaginal lining.^30^ The increase in vaginal pH decreases the levels of normal vaginal bacteria, namely *Lactobacillus*, which allows for overgrowth of other bacteria, some of which are commonly seen with UTIs. These uropathogens can then spread to and infect the bladder. Decreased estrogen can also result in shortening of the urethra and weakening of pelvic connective tissue, which contribute to the development of UI and POP, respectively.^31,32^

Vaginal estrogen is prescribed to peri- and postmenopausal women who have lower urinary tract symptoms, such as UTIs, UI, and POP.^9^ A systematic review found use of vaginal estrogen alone to be inferior to antibiotic suppression but superior to no treatment when comparing by rate of UTIs.^33^ There was also a decrease in incontinence symptoms when comparing vaginal estrogen with placebo.^33^

A study by Rahn et al. found that vaginal tissue in women undergoing hysterectomy for uterine prolapse had increased thickness and mature collagen synthesis in those treated with preoperative vaginal estrogen compared with those given a placebo.^34^ Although outcomes of the surgeries were not reported in these participants, the biopsy findings indicated that vaginal estrogen may strengthen vaginal tissue for suturing and decrease risk of POP relapse. In another study, estradiol given before prolapse surgery led to decreased incidence of cystitis a month after surgery.^35^

Although estrogen has been shown to be useful in postmenopausal women suffering from UTIs, UI, and POP, it is avoided in women with a history of estrogen-dependent breast cancer due to the risk of stimulating tumor growth. In breast cancer survivors, low doses of vaginal estrogen have been used to improve vaginal symptoms while also not significantly increasing the risk of recurrence for breast cancer.^33,36^

However, at high levels, vaginal estrogen may lead to a sustained increase in serum estrogen levels and thus act similarly to oral estrogen.^37^ Thus, vaginal estrogen is still contraindicated in this population. There is a concern that this lack of hormonal therapy negatively impacts the care of these postmenopausal women when affected by UTIs, UI, and/or POP, but the literature on this topic is scant. Similarly, not much is known regarding the long-term outcomes of using only nonhormonal treatments in this population.

Although viewed as potentially detrimental, tamoxifen, which was initially believed to be completely antiestrogenic, can produce an estrogenic effect in certain tissues, such as the bones and blood vessels.^4^ Its impact on vaginal tissue is not as well understood. Tamoxifen has been linked to worsening vulvovaginal atrophy and accompanying symptoms of vaginal irritation and dryness,^4^ indicating a reduction in estrogen, yet histologically, in postmenopausal women, tamoxifen seems to have a partial agonistic effect. Studies by Ferrazzi et al.^38^ and Boccardo et al.^39^ found an increase in the karyopyknotic index, which serves as a marker for estrogenic effect, in vaginal smears after postmenopausal participants began tamoxifen.

Another measure of estrogenic effect, epithelial cell maturation value, also increased up to 89% in Pap smears of postmenopausal women who started tamoxifen compared with before.^40,41^ Interestingly, the study by Mortimer et al.^42^ included both pre- and postmenopausal women and found the histological estrogen effect to be more prevalent in postmenopausal women but did not find a correlation with age for the vulvovaginal atrophy symptoms.

These findings suggest that there is possibly a shift in vaginal estrogen receptors with age, changing a primarily antagonistic effect to a partial or weak agonistic effect. Regardless, tamoxifen could complicate the management of UTIs, UI, and POP in these women. No studies have shown a link between tamoxifen and risk of UTIs; however, research shows that tamoxifen can increase the incidence of POP, and in one case report, tamoxifen led to the development of worsening stress UI.^8,43,44^

It is unclear how much prior tamoxifen use affects current and future surgical operation. Standard tamoxifen treatment of 5 years has shown to confer reduction of breast recurrence risk for at least 15 years after discontinuation^45^; however, little is known on the long-term effects on vaginal tissue. Ferrazzi et al.^38^ reported that vaginal smears atrophied after discontinuation of tamoxifen for 2 months, whereas Eells found that length of time on tamoxifen was not correlated with the estrogen effect on the vagina seen histologically.^40^

Despite concerns of lingering antiestrogenic effects after stopping tamoxifen, our patients with previous tamoxifen use had successful treatment of their urological conditions. Future research into whether this is true just for patients off of tamoxifen for 1–4 years (as was the case for most of our previous tamoxifen use patients) or for all time points, whether a few months or as far as 15 years after stopping tamoxifen, is needed.

Strengths of this study include access to detailed electronic medical records and long-term follow-up data. Like the few other series on this rarely reviewed topic (see Table 1), our sample size was limited even in an FPMRS specialized tertiary care center. This is a limitation that will always prevent larger studies until data as those presented in this study will encourage treating physicians and suffering women to seek care for these three common pelvic floor-related and bladder conditions.

Indeed, because all these postmenopausal women experienced symptomatic improvement at a minimum of 6 months follow-up, this real life practice experience at one tertiary center indicates that the treatment of these conditions can still be favorable and should not be denied to these women on the basis of their tamoxifen exposure and inability to use vaginal estrogens. Future studies should consider a prospective controlled multicentric approach to enroll a larger population of women in whom the impact of tamoxifen exposure can be better evaluated in terms of postsurgical pelvic reconstruction healing risks and satisfactory outcomes over time.

## Conclusions

In this limited observational series in postmenopausal women with lower genitourinary tract pathology and current or previous exposure to tamoxifen, therefore, unable to use oestrogen, their respective treatment for UTIs, UI and/or POP produced satisfactory outcomes with midterm follow-up consistent with data in women not exposed to tamoxifen and benefitting from estrogen supplementation. Therefore, the management of these women should not be hampered or altered due to the inability to use vaginal estrogens as part of their armamentarium.

## Abbreviations Used

BMI: body mass index
FPMRS: female pelvic medicine and reconstructive surgery
IIQ-7: Incontinence Impact Questionnaire
IQR: interquartile range
POP: pelvic organ prolapse
POP-Q: POP quantification
UDI-6: Urinary Distress Inventory
UI: urinary incontinence
UTI: urinary tract infection
VCUG: voiding cystourethrogram
